# A rare complication of cerebral venous thrombosis during simple percutaneous coronary intervention: A case report

**DOI:** 10.1097/MD.0000000000024008

**Published:** 2021-01-29

**Authors:** Ming Yuan Lo, Ming-Shiu Chen, Hsuan-Ming Jen, Chien-Cheng Chen, Thau-Yun Shen

**Affiliations:** a Cardiovascular Center, Show Chwan Memorial Hospital, Changhua, Taiwan; b Cardiology Department, Chang Bing Show Chwan Memorial Hospital, Lukang Town, Changhua County, Taiwan.

**Keywords:** cerebral venous thrombosis, cerebrovascular accidents, percutaneous coronary intervention, thromboembolism

## Abstract

**Rationale:**

Cerebrovascular accidents (CVAs) after percutaneous coronary intervention (PCI), although rare, are associated with high in-hospital morbidity and mortality rates. Cerebral venous thrombosis (CVT) is an uncommon cause of CVAs compared with arterial disease but is associated with favorable outcomes in most cases. We present a rare case of CVT following a simple PCI procedure with stent implantation, which has not been previously reported in the literature.

**Patient concerns:**

A 78-year-old woman with hypertension, hyperlipidemia, and coronary artery disease received simple PCI with stent implantation. After PCI, she developed a throbbing headache with nausea and vomiting, with her blood pressure increasing to 190/100 mmHg. Drowsiness, disorientation, and neck stiffness were noted. Neurological complication due to the PCI procedure was highly suspected.

**Diagnosis:**

Noncontrast brain computed tomography was performed along with emergency neurological consultation, and the patient was diagnosed as having acute CVT.

**Interventions:**

The patient was treated with anti-intracranial pressure therapy and anticoagulation therapy through low-molecular-weight heparin and was subsequently treated with warfarin.

**Outcomes:**

After treatment, the patient's symptoms and signs gradually subsided, and her clinical condition improved. She was discharged with full recovery thereafter.

**Lessons:**

A case of acute CVT, a rare, and atypical manifestation of venous thromboembolism and CVA, complicated simple PCI with stent implantation. During PCI, identifying patients with a high risk of a CVA is critical, and special care should be taken to prevent this devastating complication.

## 1 Introduction

Coronary artery disease (CAD) is among the leading causes of morbidity and mortality worldwide. Approximately 16.5 million Americans adults have CAD, with the total CAD prevalence being 6.3%.^[1]^ The main pathogenesis of CAD is the development of atherosclerotic lesions in the coronary arteries. Patients with stable CAD are often treated with revascularization through percutaneous coronary intervention (PCI), which typically involves balloon angioplasty and stent implantation. With advances in PCI technology, PCI has become one of the most common approaches adopted for patients undergoing coronary revascularization.^[2]^ In Europe, 1.5 million patients had PCI in 2010, and approximately 1.5 million people undergo PCI in the United States every year.^[3]^

Despite improvements in PCI techniques, cerebrovascular accidents (CVAs) after PCI remain one of the life-threatening adverse complications associated with both high mortality and high morbidity rates.^[4,5]^ The incidence of CVAs after PCI is low, with its rate being 0.07% to 1.4%,^[4–6]^ and the incidence of CVAs ranges from 0.18% to 0.44%,^[5,7]^ despite stroke being a common complication of cardiac surgery.^[8]^ Cerebral venous thrombosis (CVT) is an uncommon cause of CVA compared with arterial disease but is associated with favorable outcomes in most cases. Herein, we report a rare case of a patient with unexpected CVT after simple PCI with stent implantation.

## 2 Case report

A 78-year-old woman with hypertension, hyperlipidemia, and CAD received PCI with stent implantation 1 decade ago. At the time, she was taking long-term medications, including antiplatelet, antihypertensive, and statin drugs, and was under a relatively good general condition. She developed intermittent chest pain related to exertion and associated with dyspnea that persisted for approximately 2 weeks. She visited the outpatient department of a cardiovascular center and was initially prescribed extended-release nitrate and sublingual nitroglycerin. Echocardiography showed normal cardiac function without regional wall motion abnormality. Electrocardiography revealed a sinus rhythm with nonspecific ST-T change. Despite her initial treatment, the patient's symptoms persisted, and she was scheduled to receive diagnostic cardiac catheterization. Upon physical examination, the patient's appearance suggested acute illness; her blood pressure was 145/90 mmHg, heart rate was 58 beats per minute, respiratory rate was 12 breaths per minute, and body temperature was 36.5 °C. Head and neck examination yielded no notable findings, and chest examination revealed bilateral, symmetrical expansion, and mild basal rales but no wheezing. Heart auscultation indicated regular heart beats, grade I to II systolic murmurs over the left lower sternal border, and S4 gallops. The patient's abdomen was soft and supple, with no tenderness, and her lower extremities were movable, with no pitting edema. Neurological examination yielded no notable findings.

**Figure 1:**
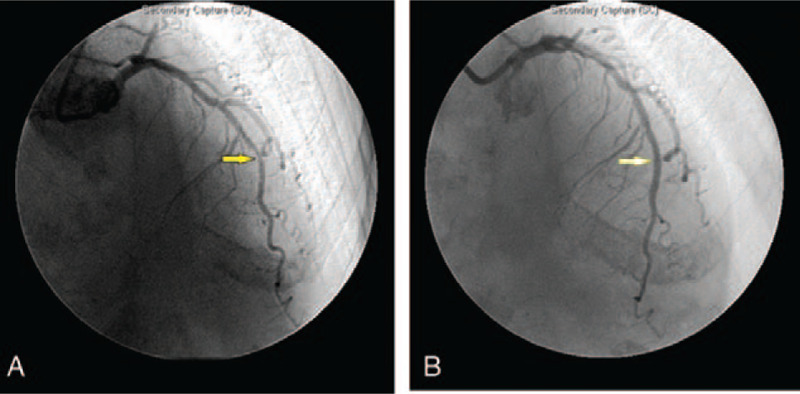
Coronary angiograph (A) before and (B) after PCI with stent implantation. PCI = percutaneous coronary intervention.

**Figure 2:**
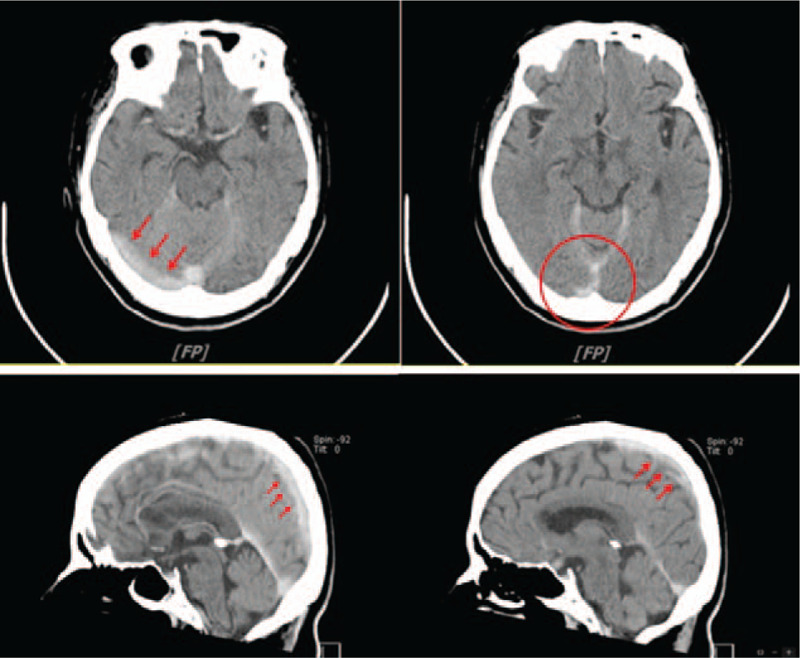
CT scans revealing CVT. CT = computed tomography, CVT = cerebral venous thrombosis.

After initial loading of dual antiplatelets, including 300 mg of clopidogrel and 100 mg of aspirin, cardiac catheterization was performed. In the distal left anterior descending artery, a discrete lesion was observed, with 80% to 85% luminal stenosis. After heparinization with 10,000 units of unfractionated heparin, successful balloon angioplasty with subsequent provisional stenting was performed with optimal angiographic results (Fig. 1). The preprocedural activated clotting time (ACT) was 156 seconds, and the postprocedural ACT was 312 seconds. The patient initially reported experiencing a headache during the procedure, and this symptom was attributed to the nitrate effect. However, the headache persisted, and her blood pressure increased to 190/100 mmHg. Nifedipine and morphine were then administered, and the patient was initially stabilized. Approximately 1 hour later, throbbing headache with nausea and vomiting developed. However, she reported no chest pain or cold sweats, and no vital sign deterioration was observed. The patient had normal muscle power with no focal signs, but drowsiness, disorientation, and neck stiffness were noted. With the suspicion of neurological complications after the PCI procedure, noncontrast brain computed tomography (CT) was performed, along with emergency neurological consultation. The patient received a diagnosis of acute CVT (Fig. 2).

The patient was transferred to the intensive care unit and treated with anti-intracranial pressure therapy and anticoagulation through low-molecular-weight heparin (LMWH). Her coagulation profile was monitored, and she was administered anticoagulation treatment with warfarin. After treatment, the patient's symptoms and signs gradually subsided. She was transferred to the general ward and discharged with full recovery thereafter. Laboratory data revealed that the patient had protein S deficiency, which may have been the cause of the CVT. The follow-up treatment regimen included antiplatelet therapy for 3 to 6 months, followed by clopidogrel alone and indefinite anticoagulation treatment with warfarin.

## 3 Discussion

CVT is a rare manifestation of venous thromboembolism (VTE) and stroke, representing only <1% of all strokes, with a prevalence of only 5 per 1 million.^[9,10]^ CVT causes dysfunction primarily through 2 mechanisms: thrombosis of the cerebral veins leads to localized edema of the brain and venous infarction, and thrombosis of the major sinuses causes intracranial hypertension, leading to increased venous pressure and impaired absorption of cerebrospinal fluid.^[11]^ The most common symptom of CVT is headache, affecting approximately 90% of patients. Isolated headache is less common but may pose a clinical challenge.^[12,13]^ Hemorrhagic conversion was reported to occur in 35% to 39% of patients.^[12]^ Brain imaging technology has been improved for the diagnosis of CVT, including conventional CT, CT venography, magnetic resonance imaging, magnetic resonance venography, and conventional angiography.^[14]^ Conventional CT and CT venography have the advantages of being widely available and enabling the prompt assessment of major sinuses; their major limitations, however, include radiation, risks related to exposure to intravenous contrast agents, and poor visualization of deep and cortical venous thrombosis.^[15]^

CVA complications occur in numerous interventions, such as PCI, thrombolytic therapy,^[16]^ and coronary artery bypass grafting (CABG).^[17]^ CVAs constitute a common complication of CABG (1%–5%), despite improvements in surgical techniques and cardioplegic agents.^[17,18]^ PCI complications are rare but potentially devastating and irreversible, with substantial impacts on patient prognosis and quality of life.

CVAs after PCI are associated with hypertension, diabetes mellitus, a CVA history, impaired renal function, lower ejection fractions, and hemodynamic instability requiring intra-aortic balloon pumping.^[5,19]^ Moreover, relevant risk factors include cardiac catheterization for urgent or emergency procedures—which may lead to further scraping of aortic plaque and increase the risk of emboli to the brain. History of application of thrombolytics and intravenous heparin, longer cardiac catheterization procedures and the use of a greater volume of contrast agents are also the risk factors to increase CVA complications during PCI.^[19]^ Similarly, the predictors of an increased risk of CVA after PCI are older age (often with a history of hypertension, diabetes mellitus, stroke, or transient ischemic attack), renal failure, intra-aortic balloon pump use, congestive heart failure, impaired left ventricular function, and angiographic disease burden.^[5,7,16,19]^ These are similar to risk factors for both hemorrhagic and nonhemorrhagic stroke in patients with ST-segment elevation myocardial infarction after thrombolytic therapy.^[20–22]^ Furthermore, studies have demonstrated that an inappropriately high dosage of antithrombotic agents may increase the risk of hemorrhagic and nonhemorrhagic stroke in patients with myocardial infarction.^[23]^ Thus, caution should be exercised when assigning a heparin dosage for patients undergoing PCI. Moreover, compared with men, women have higher rates of PCI complications, particularly bleeding complications, regardless of their age, and young women have a higher mortality risk after elective PCI. In 65% of cases in women, CVT was reported to be related to risk factors unique to women, such as oral contraceptive use, pregnancy, the puerperium, and reception of hormone therapy.^[24]^

In addition to the aforementioned risk factors, thrombophilia is a disorder in which the affected patient is at risk of thromboembolism. This type of thromboembolism generally refers to VTE, which typically occurs in patients who are older, are bed ridden, have had repeated episodes of VTE, or have an obvious family history or as a result of pregnancy or surgery. Several inherited and acquired CVT-associated thrombophilic factors have been identified. These include factor V Leiden (causing resistance to protein C), prothrombin *G20210A*, and JAK2 *V617F* mutations; protein C, protein S, or antithrombin III deficiency; increased factor VIII levels; antiphospholipid antibody syndrome (presence of anticardiolipin antibodies, antiphospholipid or anti-β2 glycoprotein antibodies, and lupus anticoagulants); PAI-1 polymorphisms; dysfibrinogenemia; and hyperhomocysteinemia.^[25–27]^ Factors associated with mild thrombophilia include dysfibrinogenemia and heterozygous factor V Leiden and prothrombin *G20210A* mutations. Those associated with severe thrombophilia are deficiency of protein C, protein S, or antithrombin III; the presence of antiphospholipid antibodies; homozygous factor V Leiden mutation; and multiple abnormalities. In a follow-up study of participants with CVT, a 4-fold higher incidence of deep venous thrombosis or pulmonary embolism was reported in patients with severe thrombophilia compared with those without thrombophilia.^[28]^ Therefore, our patient with severe thrombophilia had numerous predisposing factors.

Patients with CVAs after PCI have been noted to have increased rates of in-hospital complications, such as acute renal failure, procedural failure, myocardial infarction, severe hemodynamic disturbance, emergency CABG, and access-site complications.^[5,6,19]^ All deaths in these patients are caused by ischemic or hemorrhagic stroke, which has high in-hospital mortality rates (20%–37%).^[6,7,19]^ Patients with CVAs after CABG were reported to have a significantly increased length of hospital and intensive care unit stay and a 5-fold higher rate of in-hospital mortality compared with patients without CVAs.^[17,29]^ CVAs after PCI are associated with relatively high rates of in-hospital mortality and morbidity, emphasizing the need for timely recognition and prevention of risk factors predisposing patients to this complication.

The development of evidence-based treatment is limited by the low incidence of CVA after PCI; no guidelines have been developed for a single therapeutic approach. Therefore, all recommended treatments are mainly based on expert opinions and small case series.^[30]^ Thrombolytic therapy and mechanical embolectomy are the currently used treatment approaches. Furthermore, pharmaceutical agents, such as gangliosides, glutamate receptor antagonists, and antioxidants, may minimize neuronal damage and reduce the occurrence of stroke.^[31]^ For patients with CVT, initial anticoagulation is the standard treatment; it prevents thrombus growth, facilitates recanalization, and prevents other thrombotic events, including deep vein thrombosis and pulmonary embolism.^[27]^ Coutinho et al^[32]^ reported that LMWH is possibly safer and probably more efficacious than unfractionated heparin. Different endovascular approaches may be applied for patients with CVT developing progressive neurologic deterioration despite receiving intensive medical treatment such as anticoagulation therapy. Thrombolysis and mechanical thrombectomy are considered potential alternative treatments for improving early recanalization in patients for whom anticoagulation treatment is unsuccessful.^[27]^

Mortality is lower in patients with CVT than in those with arterial stroke. Over time, the mortality rate of CVT has significantly decreased; possible explanations include improvements in treatment, a shift in risk factors, and most notably, an increase in the number of mild cases identified by using advanced diagnostic technologies. The current reported mortality rate is 2% to 38%.^[33–36]^ Most patients with CVT have a good prognosis. Approximately 80% of patients with CVT have a modified Rankin Scale (mRS) score of 0 or 1, but they usually have residual symptoms and are often unable to return to their previous jobs. The patients have National Institutes of Health Stroke Scale scores of ≥2, and a low educational level affects both functional recovery and unemployment.^[37]^ In CVT, prognosis is not associated with a hypercoagulable state, the number of involved venous sinuses, or the presence of intracranial hemorrhage or seizures.^[38]^

CVT as an extremely rare complication of PCI has not been reported in the literature. In our case, timely diagnosis and prompt initiation of anticoagulation treatment were crucial for resolving severe signs and stabilizing the patient's neurological deterioration.

## 4 Conclusion

During PCI, identifying patients with CVA risk factors is crucial, and particular care should be taken to prevent this devastating complication. Although CVT is an uncommon cause of CVAs and an extremely rare complication of PCI, it is a potentially serious and life-threatening condition that necessitates early detection and prompt treatment.

## Author contributions

**Conceptualization:** Ming Yuan Lo, Ming-Shiu Chen, Thau-Yun Shen.

**Writing – original draft:** Ming Yuan Lo, Ming-Shiu Chen, Thau-Yun Shen.

**Writing – review & editing:** Hsuan-Ming Jen, Chien-Cheng Chen, Thau-Yun Shen.

## Abbreviations

Abbreviations: ACT = activated clotting time, CABG = coronary artery bypass grafting, CAD = coronary artery disease, CT = computed tomography, CVA = cerebrovascular accident, CVT = cerebral venous thrombosis, IABP = intra-aortic balloon pumping, LMWH = low-molecular-weight heparin, MI = myocardial infarction, MRI = magnetic resonance imaging, MRV = magnetic resonance venography, PCI = percutaneous coronary intervention, TIA = transient ischemic attack, VTE = venous thrombo-embolism.
